# Underestimation of smoking hazards and smoking cessation intervention efficiency among healthcare professionals: A cross-sectional study among Korean occupational health nurses

**DOI:** 10.18332/tid/162320

**Published:** 2023-05-05

**Authors:** Jihye Lee, Saerom Lee, Woncheol Lee, Seung Hyeun Lee, Won Gun Kwack, Young Joong Kang

**Affiliations:** 1 Occupational Safety and Health Research Institute, Korea Occupational Safety and Health Agency, Ulsan, Republic of Korea; 2 Korean Industrial Health Association, Seoul, Republic of Korea; 3 Department of Occupational and Environmental Medicine, Kangbuk Samsung Hospital, School of Medicine, Sungkyunkwan University, Seoul, Republic of Korea; 4 Division of Pulmonary, Allergy, and Critical Care Medicine, Department of Internal Medicine, Kyung Hee University School of Medicine, Seoul, Republic of Korea; 5 COMWEL Incheon Hospital, Korea Workers’ Compensation and Welfare Service, Incheon, Republic of Korea

**Keywords:** cancer, cigarette smoking, occupational health nurses, smoking cessation, workplace smoking cessation

## Abstract

**INTRODUCTION:**

Occupational health nurses (OHNs) in South Korea who visit the workplace periodically could play a key role in smoking cessation. It would be helpful to assess their understanding of smoking hazards and smoking cessation methods to encourage them to provide smoking intervention services at the workplace. This study aimed to investigate the knowledge of smoking hazards and perceptions of smoking cessation methods among OHNs.

**METHODS:**

We conducted an anonymous self-administered cross-sectional questionnaire survey of 108 OHN nurses employed in an occupational health service outsourcing specialized agency with 19 regional branches in Korea from July to August 2019. We assessed the perceptions of the OHNs about smoking interventions, hazards of smoking, and perceived competence to counsel smokers according to training experience, using chi-squared tests and Fisher’s exact tests.

**RESULTS:**

The majority of the nurses underestimated the smoking-attributable fraction for lung cancer (78.7%), chronic obstructive pulmonary disease (64.8%), and mortality (49.0%), regardless of training experience on smoking cessation, while more than half perceived their skill and knowledge to counsel patients concerning smoking as inadequate (56.5%). However, those trained in smoking cessation interventions felt more competent in smoking cessation counselling, with 52.2% and 29.3% in the trained and non-trained groups, respectively (p=0.019).

**CONCLUSIONS:**

The OHNs in this study underestimated smoking hazards and perceived themselves as lacking counselling skills regarding smoking cessation interventions. It is necessary to encourage OHNs to promote smoking cessation by increasing their knowledge, skills and competence in smoking cessation interventions.

## INTRODUCTION

Occupational health nurses (OHNs) are specialized nurses in the care and well-being of people at work. They have direct contact with employees and are often approached with health-related questions and problems. The UK National Health Service (NHS) suggests that employees often see their OHNs as a ‘first port of call’ and seek advice on various health and safety-related matters, including work and non-work related conditions^1^. OHNs can act as smoking cessation advisors in workplaces. The workplace appears to be a useful setting to help people quit smoking if it has an adequate professional anti-smoking program^2^. Previous studies have suggested that OHNs need to be provided with opportunities to increase their smoking cessation intervention techniques and their roles as smoking advisors to promote occupational health in small- and medium-sized enterprises (SMEs) in Korea^3^.

In the Republic of Korea, The Enforcement Decree of the Occupational Safety and Health Act (OSH Act) allows SMEs to outsource occupational health management activities of their employees to occupational health services outsourcing specialized agencies (OHSO SAs) designated by the Ministry of Employment and Labor^4^. The OHNs in the OHSO SAs in Korea took over the employers’ legal obligation to protect SMEs’ employee health in accordance with the Enforcement Decree of the OSH Act^5^. OHNs visit SMEs monthly for occupational health management, health consultations, and healthcare education^6^. In 2016, 114 SAs facilitated the occupational health management activities of approximately 28000 SMEs^4^.

The OHNs in OHSO SAs are closely associated with SMEs in Korea, and they have the potential to deliver sufficient support and advice for smoking cessation promotion in the workplace. Previous studies for workplace health promotion suggest that the workplace is a setting where large groups of smokers can potentially be reached for health promotion^2^ and a setting for changing the perception of smoking and reducing productivity losses and medical expenses with an entire workplace smoking cessation program^7^. In addition, workplace smoking cessation programs can help smokers with lower motivation levels to quit and prevent any smoking relapses^8^. Furthermore, a previous study of Korean workplace conditions revealed that small enterprises with <50 employees had the highest smoking rates, 56.2% among male workers, those with 50–299 employees had a rate of 53.5%, and those with ≥300 employees had a rate of 45.5% among male smokers^9^. They suggested that this difference was related to the lack of smoking cessation policies in smaller workplaces. The lack of resources and expertise is the main barrier towards adopting health promotion programs at smaller workplaces^10^. Therefore, it could be meaningful to delegate the role of promoting smoking cessation to OHNs who are in close contact with the workplaces in SMEs. However, a previous study suggested that nurses in Korea hardly participate in smoking cessation counselling and do not have the opportunity to develop the skills to counsel smoking cessation^11^. Despite the efficacy of nursing intervention for tobacco cessation, lack of appropriate knowledge and/or skill presents a major problem for implementation^12^. A study amongst Hong Kong nurses also revealed that many are not yet well prepared to intervene in tobacco cessation and that it might be due to insufficient opportunities to develop their skills for counselling smoking cessation^13^.

It is necessary to verify the skills and knowledge of OHNs before the assignment of a role in the counselling of smoking cessation, in order to achieve successful outcomes. Thus, it is important to ascertain the perceptions and knowledge of OHNs on smoking cessation interventions. It should come as no surprise that cigarette smokers develop lung cancer and chronic obstructive pulmonary disease (COPD), the two leading causes of death^14^, but it is a separate issue regarding how seriously and accurately smoking is considered a hazard. We aimed to investigate OHNs’ perceptions and awareness concerning smoking and assess whether their training experience in smoking cessation could influence these.

## METHODS

### Study participants and procedure

We invited all OHNs employed in an occupational healthcare corporation with 19 regional OHSO SA branches nationwide to participate in this study. In this occupational healthcare corporation, 150 OHNs were employed to provide occupational health services to SMEs of various industries with 50–299 employees. We contacted the 150 OHNs via e-mail and conducted a questionnaire survey from July 2019 to August 2019. The e-mail contained a link to an informed consent form and a questionnaire. In total, 110 out of 150 OHSO nurses responded to the questionnaire; two subjects with inconsistent responses were excluded, and a total of 108 subjects were selected as final study subjects. The creation of the questionnaire and collection of completed questionnaires were performed using an online survey software program (SurveyMonkey, San Mateo, CA, USA). The survey was conducted anonymously.

### Measures

This study utilized a previous questionnaire developed and piloted by Raupach et al.^15^ to investigate medical students’ knowledge about smoking-related mortality, health risks, and the effectiveness of various smoking cessation methods, and also to analyze related general information and smoking status.

In order to conduct a survey targeting Korean OHNs, the question related to the medical school curriculum was removed, and a question about smoking status was modified and classified as current smoker, past smoker, or never smoked. The two dichotomous questions about whether respondents knew any smoker/non-smoker older than 90 years from the original study were not utilized. Several questions were added to determine the effect of various characteristics on their perception of smoking. The information obtained included sex, age, and the years of working experience as a nurse and as an OHN. We also included a question about whether OHNs had received training for smoking cessation interventions. The modified questionnaire was translated from the original English into Korean and back to English, and linguistic inconsistencies were revised.

The questionnaire asking about smoking hazards and smoking method efficiency consisted of two open-ended questions, three 11-point Likert scale (0–100%) questions, one 4-point question and a dichotomous question. The two open-ended questions regarding smoking-related toxicity and related death toll were used: ‘In your opinion, what component of tobacco smoke is mainly responsible for the increased risk of coronary artery disease among smokers? (multiple responses allowed)’ and ‘Please estimate how many people die of smoking-related diseases in Korea annually’. We referred to one of the most recent studies regarding the smoking-attributable death toll among Korean adults^16^. In particular, it was reported that there were approximately 60000 smoking-related deaths in 2012, and that the number of deaths was expected to decrease as the adult smoking rates decreased. The following three 11-point Likert scale (0–100%) questions asked OHNs to estimate the smoking-attributable fractions (i.e. percentage of all cases of a specific disease caused by smoking) for lung cancer, COPD and smoking-related disease mortality for smokers using an 11-point scale (0–100%) to assess their perception of smoking hazards. According to previous studies^17^, the smoking-attributable fractions for COPD and lung cancer were approximately 80–90% and 81–95%, respectively. Therefore, we regarded the ratings of <80–90% as answers that underestimated smoking-attributable health risks and following the results of the British physicians, half to two-thirds of smokers who smoke one pack a day or more will die from a smoking-related disease^18^; thus, the rating of 50–70% for smoking-related disease mortality was considered as the criterion to evaluate the underestimation of smoking hazards in this study. A question asked OHNs’ perceived effectiveness of several smoking cessation methods (i.e. willpower alone, advice from a general physician, pharmacotherapy [nicotine replacement therapy; NRT], smoking cessation program with the use of an anti-smoking drug, self-help material, and acupuncture) and was determined using a 4-point Likert scale ranging from ‘very effective’, ‘effective’, ‘slightly effective’, to ‘not effective’. The OHNs were informed to consider it to be ‘very effective’ if the continuous abstinence rate was at least 30% after one year, according to the overall findings from previous studies^18,19^. The pharmacotherapy and anti-smoking drug in the smoking cessation methods was NRT originally. It was modified because the National Health Insurance Service (NHIS) in Korea covered fees for both consultation and smoking cessation aids, including not only NRT products but also varenicline and bupropion for voluntary smoking cessation clinic visits across the country since 2015^20^. A dichotomous question asked OHNs whether they felt competent in giving advice to those seeking help to quit smoking.

### Statistical analysis

The data were analyzed using SPSS version 21.0 (IBM Corp., Armonk, NY, USA). The level of significance was set at p<0.05. The frequency analysis of training experience for smoking cessation interventions was conducted according to the demographic characteristics of the participants. The differences in training experience for smoking cessation interventions according to sex, age, knowledge, and smoking behavior of family members were investigated using chi-squared tests. Statistically significant differences in training experience with respect to years of working experience as a nurse and as an OHSO OHNs were evaluated using an independent t-test. Frequency distributions were used to describe the responses of OHSO nurses to the open-ended question about the components of tobacco smoke related to increased coronary artery diseases among smokers and the question asking about their perception of the effectiveness of several smoking cessation methods. Chi-squared tests were used to determine the differences in smoking cessation education and categorical variables, such as number of smoking-attributable deaths and smoking-attributable fractions of mortality, lung cancer, and COPD. If the expected frequencies were too low (>20% of the cells had an expected count of <5), Fisher’s exact tests were used instead of the chi-squared tests. The open-ended question estimating smoking-attributable deaths in South Korea were equally categorized into five levels between 0 and 100000, and the differences in smoking cessation education and categorical variables were analyzed using the chi-squared test.

## RESULTS

### Demographics and occupational characteristics

The demographic characteristics of participants, according to having and not having training experience for smoking cessation interventions, are presented in Table 1. All the 108 participants were female and non-smokers. The proportion of those who answered that they felt having appropriate expertise to help smokers quit smoking was 52.2% (n=35) and 29.3% (n=12) in the trained and non-trained groups, respectively (p=0.019).

**Table 1:** Demographic and occupational characteristics of Korean OHNs according to training experience for stop-smoking intervention (N=108)

*Characteristics*	*OHNs with training experience n (%)*	*OHNs without training experience n (%)*	*p*
**Sex**			
Female	67 (100)	41 (100)	
**Age** (years)			
<30	6 (9.0)	5 (12.2)	0.005*
30–39	25 (37.3)	23 (56.1)	
40–49	18 (26.9)	12 (29.3)	
≥50	18 (26.9)	1 (2.4)	
**Years of experience as OHN,** mean ± SD	9.82 ± 6.48	7.08 ± 4.61	0.020**
**Years of experience as nurse,** mean ± SD	15.64 ± 7.98	12.53 ± 5.63	0.032**
**Do you think you have the expertise to help smokers who want to quit smoking?**			
Yes	35 (52.2)	12 (29.3)	0.019***
No	32 (47.8)	29 (70.7)	

OHN: occupational health nurse. SD: standard deviation.

* Fischer’s exact test.

** Student’s t-test.

*** Chi-squared test.

### Tobacco toxicity

**Figure 1:**
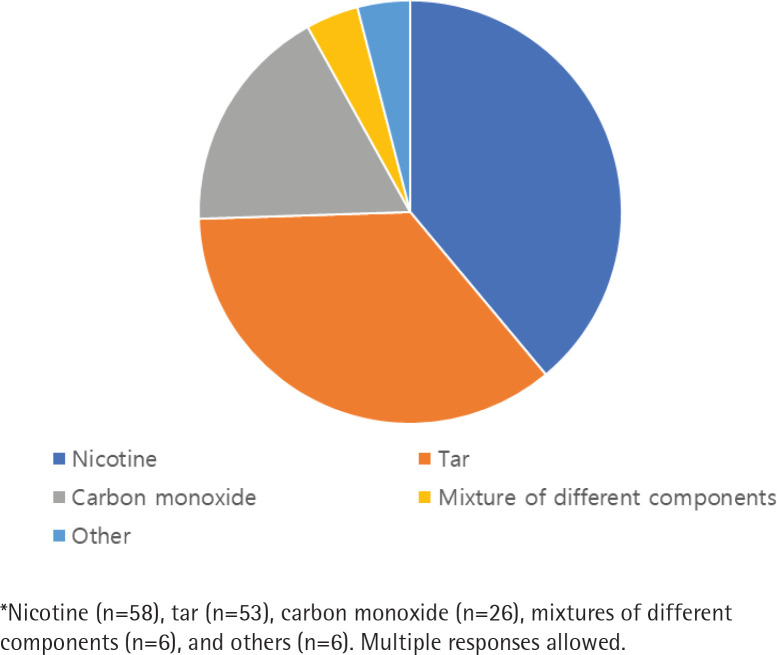
Answers* of OHNs to the question on which of the components of tobacco they considered cause coronary artery disease

Among the 108 respondents, 99 nurses gave 149 collectible answers with multiple responses to the open-ended question regarding the components in tobacco smoke responsible for the increased risk of coronary artery disease among smokers (Figure 1). Although multiple responses were possible, approximately 30% of nurses (n=32) blamed nicotine alone for coronary heart disease, and 20% (n=24) mentioned tar as the component solely responsible for its etiology. Nicotine was the most commonly mentioned component (58 answers), followed by tar (53 answers), carbon monoxide (26 answers), mixtures of different components (6 answers), and others (6 answers).

### Smoking-related deaths in Korea

The answers to ‘Please estimate how many people die of smoking-related diseases in Korea annually’ are presented in Table 2. Approximately one-third of participants chose 40001–60000 annual deaths. The proportion of those whose answer was within this range was higher in the non-trained (41.5%, n=17) than in the trained group (29.9%, n=20) but without a statistically significant difference. The proportion of respondents who underestimated smoking-related mortality was 58.2% (n=39) and 75.7% (n=31) in the trained and non-trained groups, respectively (no significant difference).

**Table 2:** Estimates by OHNs of the number of smoking-attributable deaths per year in Korea, according to training experience for stop-smoking intervention (N=108)

*Estimate*	*OHNs with training experience (N=67) n (%)*	*OHNs without training experience (N=41) n (%)*	*p*
0–20000	10 (14.9)	4 (9.8)	0.402*
20001–40000	9 (13.4)	10 (24.4)	
40001–60000	20 (29.9)	17 (41.5)	
60001–80000	11 (16.4)	5 (12.2)	
80001–100000	17 (25.4)	5 (12.2)	

* Fischer’s exact test.

### Smoking-attributable mortality and morbidity

**Figure 2:**
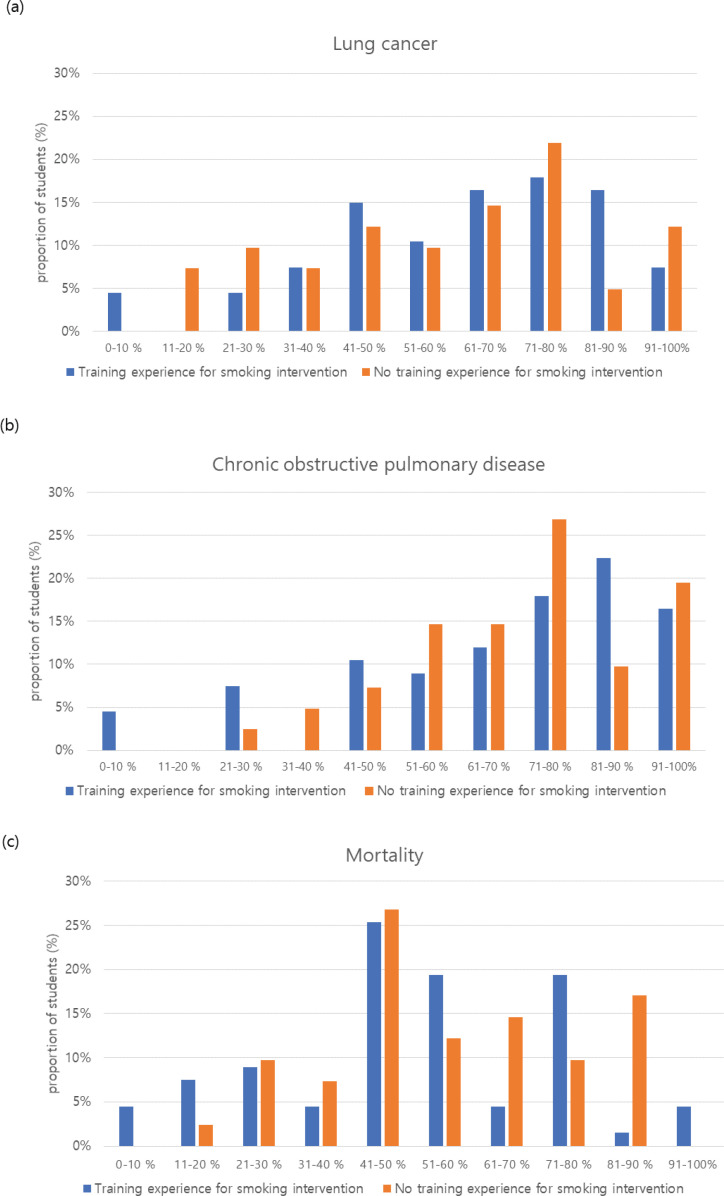
Estimates by OHNs of the smoking-attributable percentages of: a) lung cancer, b) chronic obstructive pulmonary disease, and c) mortality

Figure 2 shows nurses’ estimates of smoking-attributable fractions of lung cancer, COPD, and smokers’ deaths from smoking-related diseases. At least three-fifths of the respondents underestimated the hazards of smoking and thought that smoking was responsible for <70% of lung cancer cases, while two-fifths of the respondents thought that smoking was responsible for <70% of COPD cases. Only 16.4% (n=11) of the trained nurses and 4.9% (n=2) of the non-trained nurses answered that the smoking-attributable fraction of lung cancer was 80–90%. Moreover, only 22.4% (n=15) and 9.8% (n=4) of those in the trained and untrained groups, respectively, answered that the smoking-attributable fraction of COPD was 80–90%. These differences between the two groups were not statistically significant. Concerning the proportion of smokers who died because of smoking-related diseases, the rating of 50–70% was considered reasonable for the smoking-attributable mortality in this study. Only approximately a quarter of OHNs mentioned this range. The proportion of respondents who underestimated smoking-attributable mortality as <50% was 50.9% (n=34) in the trained group and 46.3% (n=19) in the non-trained group. The proportion of respondents whose answers were within this range was inversely higher in the non-trained (26.8%, n=11) than in the trained group (23.9%, n=16).

### Effectiveness of the smoking cessation methods

**Figure 3:**
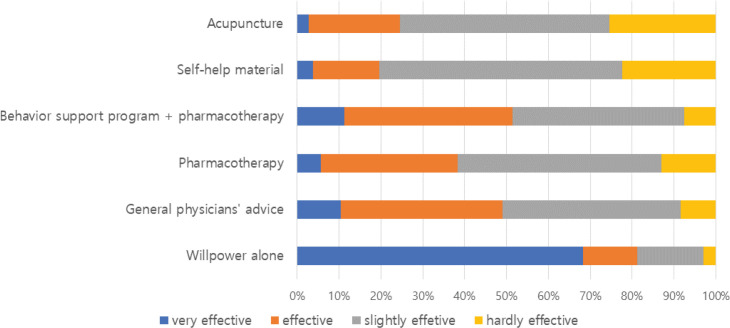
Perceptions of OHNs regarding the long-term effectiveness of different approaches to smoking cessation

The OHNs were asked to share their thoughts regarding the effectiveness of smoking cessation methods (Figure 3). The majority of OHNs answered that ‘will power alone’ is the most effective method to quit smoking, and a total 81.2% (n=82) OHNs regarded it as a very effective or effective cessation method. Regarding proven smoking cessation methods to increase the likelihood of smoking cessation, such as behavioral support program with pharmacotherapy and advice from general physicians, only half of the participants considered that these methods were very effective or effective, 51.4% (n=55) and 49.1% (n=52), respectively. In contrast, the effectiveness of acupuncture, which appears to have no scientific evidence, was considered to be very effective or effective by approximately 24.5% (n=26) of the OHNs. These OHNs’ tendencies to underestimate proven smoking cessation methods and overestimate ‘will power alone’ and ‘acupuncture’ as effective smoking cessation methods were sustained, despite smoking cessation training experiences (Supplementary file).

### Perceived competence to counsel smokers

**Figure 4:**
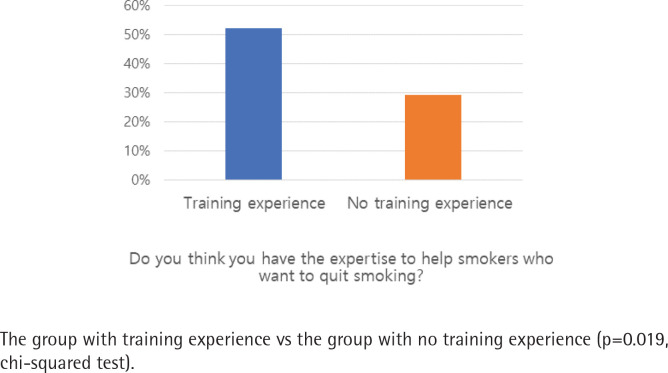
Proportion of OHNs with the expertise to help smokers who want to quit smoking

Participants were asked: ‘Do you think you have the expertise to help smokers who want to quit smoking?’ (Figure 4). More than half (59.3%, n=61) of the OHNs perceived their level of skills and knowledge to counsel patients concerning smoking as inadequate; however, the OHNs trained on smoking cessation interventions had greater confidence in smoking cessation counselling. The proportions of respondents who answered ‘yes’ to this question were 52.2% (n=35) and 29.3% (n=12) in the trained and non-trained groups, respectively, with a statistically significant difference (p=0.019).

## DISCUSSION

This study investigated the perception of OHNs regarding smoking hazards as well as the effectiveness of smoking cessation methods by asking them to estimate the risk of smoking and the effectiveness of these methods. We also analyzed the associated factors influencing the awareness of OHNs regarding the smoking hazards and perceived competence towards smoking cessation. More than half of the OHNs in this study thought that smoking was responsible for <70% of lung cancer and COPD cases or smoking was responsible for <50% of smoking-related disease mortality. This is a much-underestimated answer, compared to the smoking-attributable fractions for COPD and lung cancer being reported as approximately 80–90% and 81–95%, respectively, from previous studies^17^, and that half to two-thirds of smokers who smoke ≥1 pack a day will die from a smoking-related disease^19^.

Their knowledge of the effectiveness of smoking cessation methods was also insufficient compared to reported literature; given that an unassisted quit rate was reported at only 2–3% in the literature^21^, the majority of OHNs regarded ‘will power alone’ as very effective and more effective than the scientifically proven and recommended methods, such as physicians’ advice and pharmacotherapy with behavioral support^21-23^. Acupuncture, which appears to have no scientific evidence in previous work^24^, was overestimated as effective as pharmacotherapy, and even previous training experience on smoking cessation did not improve acknowledgement of the importance of smoking cessation methods. This underestimation of physicians’ role in smoking cessation interventions and proven smoking cessation methods can adversely affect smoking cessation attempts and policies. The OHNs with these misconceptions would be passive in referring smokers to smoking cessation intervention clinics and fail to provide smokers with proper smoking cessation information and aids.

In addition, the OHNs in our study also had misunderstandings about the harmful substances of cigarettes. Many OHNs believed that nicotine is mainly responsible for coronary artery diseases. These misunderstanding and lack of knowledge is noteworthy given the counselling role of Korean OHNs in SMEs, although this misunderstanding has been consistent also in previous studies^15,18^. Obtaining nicotine from NRT has the potential to assist smoking cessation attempts with fewer adverse events and can reduce daily cigarette consumption^25^. If OHNs regard smoking and nicotine synonymously within the context of abusing agents and cardiovascular risk factors, they would not recommend NRT to smokers and the OHNs who value unassisted willingness would be passive in referring smokers to smoking cessation intervention clinics. They might fail to provide smokers proper smoking cessation information and aids.

The estimated number of smoking-attributable deaths per year and knowledge of the overall health risk of smoking were poor in our study. If these two fatal diseases (lung cancer and COPD) were caused by smoking, deaths could be attributed to smoking. It could be stated that the OHNs in our study did not regard smoking as a serious health risk and tended to underestimate the actual severity of smoking. Moreover, their training experience for smoking intervention did not affect these results.

More than half of the OHNs believed that they did not have adequate skills and knowledge to counsel patients about smoking. Furthermore, many OHNs never had specific education on smoking cessation and had never been encouraged to undergo such training. The issues regarding the lack of counselling knowledge concerning smoking and the necessity of creating training opportunities regarding smoking cessation have been raised in previous studies^15,18,26^. Our results showed that nurses with training on smoking cessation interventions felt more competent in smoking cessation counselling, although the training experience did not make a significant difference in the OHNs’ perception of smoking hazards. Notably, educational programs for nurses in supporting smoking cessation efforts could encourage more nurses to engage in smoking cessation interventions^27^; however, if healthcare professionals had incorrect background knowledge and perceptions regarding smoking cessation or smoking hazards, ineffective methods would be recommended.

Previous studies have suggested that the workplace setting offers an opportunity to recruit large numbers of smokers while having a comparable effect with other settings such as clinic^28^ and the workplace is a favorable setting for smoking cessation because smokers’ social environment, particularly colleagues, are strongly associated with success in quitting^29^. The workplace smoking cessation programs show encouraging results, but the lack of resources and expertise is the main barrier towards adopting health promotion programs at smaller workplaces^10^. OHSO SA health management can be properly utilized for smoking cessation programs at SMEs. However, they can only passively conduct smoking cessation interventions unless they are prepared and equipped to provide qualified services. However, if OHNs who are well-prepared to deliver qualified help could advise workplace smokers during their periodic health consultation, it would be sufficient for the smoking cessation program at the workplace. Our research can serve as a basis for creating awareness about such high-quality smoking cessation services among nurses in the workplace or the general community.

To the best of our knowledge, this study is the first to report how OHNs, who visit the workplace regularly, underestimate smoking hazards and proven smoking cessation aids. Our findings also indicate that the experience of nurses in smoking cessation education made a difference only in their competence in smoking cessation counselling. We asked the OHNs to estimate objective values related to smoking hazards from recent research, and the estimated answers were derived from their knowledge concerning smoking hazards.

### Limitations

As this study was the first attempt to investigate the background perception of nurses regarding smoking cessation promotion, it has some limitations. First of all, our study was conducted based on very limited conditions to be able to be generalized. We utilized the questionnaire from a previous study which did not inform on the reliability and validity assessment of the questionnaire. It was uncertain if the participants were evaluated on their perception and knowledge of smoking-related issues by their answers. The participants were also limited, all being OHNs employed in a single OHSO SA in Korea. There is a potential bias and uncertainty of generalization of the results to other nurses and OHNs in Korea. Subsequently, to generalize the research results, it is necessary to conduct further questionnaire evaluations of subject groups. Third, despite using data from widely cited research in the field of smoking and related diseases, some referenced studies are relatively old, and there may be differences in specific values related to the smoking-attributable fraction of diseases, mortality, and the effect of anti-smoking aids and treatments. Finally, the nurses’ experience in smoking intervention was assessed only by self-reports, potentially rendering our results less reliable. However, we found that the experience of smoking cessation promotion education could make a difference in self-competence about smoking cessation promotion.

## CONCLUSIONS

This study aimed to investigate OHNs’ perception and knowledge concerning smoking and assessed whether their training experience in smoking cessation could influence their perception and awareness. The OHNs underestimated smoking hazards, and the effectiveness of various smoking cessation methods; their smoking cessation intervention training experience made them feel more confident in smoking cessation counselling but their perception regarding smoking was not significantly different. There is a need for proper education on smoking cessation for the OHNs within smoking cessation programs at SMEs in Korea. When OHNs have the right knowledge about the smoking hazards and smoking cessation methods, and have confidence based on appropriate skills in smoking cessation promotion, OHNs would be able to effectively contribute to workplace smoking cessation interventions.

## Data Availability

The data supporting this research are available from the authors on reasonable request.
