# Supplementary material for: Underestimation of smoking hazards and smoking cessation intervention efficiency among healthcare professionals: A cross-sectional study among Korean occupational health nurses
Source: Tob Induc Dis. 2023 May 5;21:55. doi: 10.18332/tid/162320 (PMC10161686; doi:10.18332/tid/162320)
Supplement: Supplementary file 1 [file TID-21-55-s1.pdf]

Supplement questionnaire

Demographic characteristics

1. Sex

a) Male b) Female

2. Age

( )

3. How many years have you worked at the current institution? (Not shown in the table)

( )

4. How many years have you worked as an occupational health nurse?

( )

5. What is your total experience as a nurse?

( )

6. Are you currently a smoker?

a) Yes

b) No, I have never smoked.

c) Past smoker, no smoking since ( ) years ago

7. Is your spouse a smoker?

a) Yes

b) No, they have never smoked.

c) Past smoker, no smoking since ( ) years ago

d) I am single

8. Is your father a smoker?

a) Yes

b) No, he has never smoked.

c) Past smoker, no smoking since (   ) years ago

Questions regarding smoking toxicity and smoking attributable morbidity

1. Please estimate how many people die of smoking-related diseased in Korea annually.

   a) 0-20,000
   b) 20,001-40,000
   c) 40,001-60,000
   d) 60,001-80,000
   e) 80,001-10,000

2. What components of tobacco smoke is mainly responsible for the increased risk of coronary artery disease among smokers? (__________________________)

3. Please estimate the proportion of cigarette smokers who die due to smoking-related diseases.

   (0-100%)

4. Please estimate the percentage of all cases of lung cancer caused by smoking.

   (0-100%)

5. Please estimate the percentage of all cases of chronic obstructive lung diseases (COPD) caused by smoking.

   (0-100%)

Questions regarding perceived effectiveness of several smoking cessation methods

6. Please estimate the long-term effectiveness of different approaches to smoking cessation.

   A. Willpower alone

① very effective ② effective ③ slightly effective ④ hardly effective

B. Advice from a general physician

① very effective ② effective ③ slightly effective ④ hardly effective

C. Nicotine replacement therapy (NRT)

① very effective ② effective ③ slightly effective ④ hardly effective

D. Smoking cessation program with an anti-smoking drug

① very effective ② effective ③ slightly effective ④ hardly effective

E. Self-help material

① very effective ② effective ③ slightly effective ④ hardly effective

F. Acupuncture

① very effective ② effective ③ slightly effective ④ hardly effective

Dichotomous questions.

1. Do you personally know a 90-year-old lifelong smoker

a) Yes b) No

2. Do you personally know a 90-year-old lifelong nonsmoker?

a) Yes b) No

3. Have you ever received any specialized training in smoking hazards and smoking cessation treatment?

a) Yes b) No

4. Do you think you have the level of expertise to help smokers who want to quit smoking?

a) Yes b) No

Supplement Figure. Perception of OHNs regarding the long-term effectiveness of different approaches to smoking cessation classified by OHNs' training experience of smoking cessation intervention; (a) OHNs with training experiences, (b) OHNs without training experiences. OHNs rated effectiveness using a 4-point Likert scale. Blue bars, very effective; orange bars effective; gray bars, slightly effective; yellow bars, hardly effective.

(a)

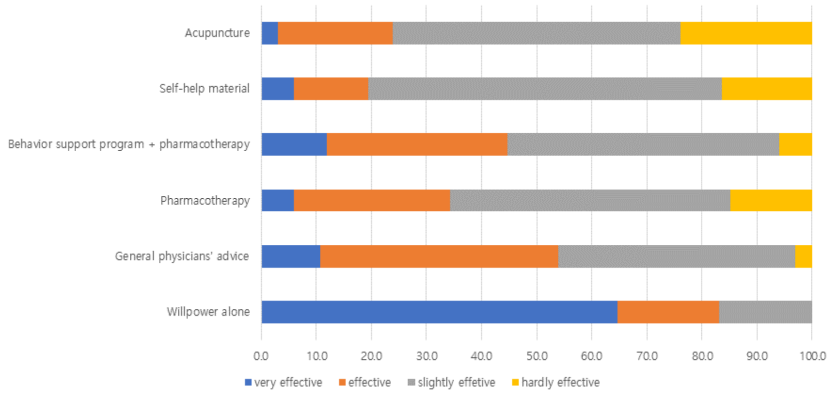

• Supplement Figure.

(b)

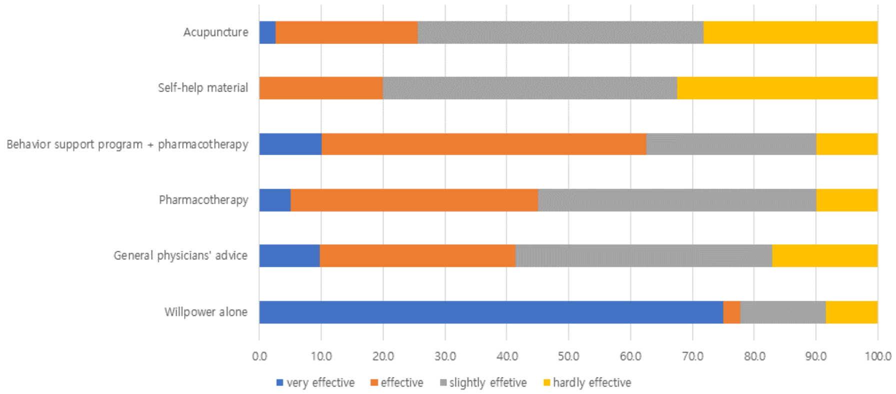

• Supplement Figure.

© 2023 Lee J. et al.
